# Novel Butane-Oxidizing Bacteria and Diversity of *bmoX* Genes in Puguang Gas Field

**DOI:** 10.3389/fmicb.2018.01576

**Published:** 2018-07-17

**Authors:** Yue Deng, Chunping Deng, Jinshui Yang, Baozhen Li, Entao Wang, Hongli Yuan

**Affiliations:** ^1^State Key Laboratory of Agrobiotechnology, College of Biological Sciences, China Agricultural University, Beijing, China; ^2^Departamento de Microbiología, Escuela Nacional de Ciencias Biológicas, Instituto Politécnico Nacional, Mexico City, Mexico

**Keywords:** butane-oxidizing bacteria, DNA-SIP, real-time quantitative PCR, *bmoX* gene, light hydrocarbon microseepage

## Abstract

To investigate the diversity of butane-oxidizing bacteria in soils contaminated by long-term light hydrocarbon microseepage and the influence of butane on the soil microbial community, a quantitative study and identification of butane-oxidizing bacteria (BOB) in soils at the Puguang gas field were performed by DNA-based stable isotope probing (DNA-SIP). For the first time, two phylotypes corresponding to the genera *Giesbergeria* and *Ramlibacter* were identified as being directly involved in butane oxidation, in addition to the well-known light hydrocarbon degrader *Pseudomonas*. Furthermore, *bmoX* genes were strongly labeled by ^13^C-butane, and their abundances in gas field soils increased by 43.14-, 17.39-, 21.74-, and 30.14-fold when incubated with butane for 6, 9, 12, and 14 days, respectively, indicating that these *bmoX-*harboring bacteria could use butane as the sole carbon and energy source and they play an important role in butane degradation. We also found that the addition of butane rapidly shaped the bacterial community and reduced the diversity of *bmoX* genes in the gas field soils. These findings improve our understanding of BOB in the gas field environment and reveal the potential for their applications in petroleum exploration and bioremediation.

## Introduction

The MPOG is a surface geochemical exploration method based on the microseepage theory that light hydrocarbons (such as C_1_–C_4_ alkanes) penetrate and migrate upward to the surface from subsurface oil and gas accumulations and that these hydrocarbons are utilized by alkane-oxidizing bacteria in the surface soil (Dayal et al., 2014). These bacteria are mostly enriched due to the continuous supply of hydrocarbon gases (Rasheed et al., 2013). Therefore, the anomalous distribution and activity of light hydrocarbons (C_1_–C_4_ alkanes) degraders can serve as an indicator for oil and gas deposits (Veena Prasanna et al., 2013; Rasheed et al., 2017). Although the distribution, abundance, and community composition of methanotrophs are well-studied in oil and gas fields (Zhang et al., 2010; Kadnikov et al., 2012; Xu et al., 2013b), the anomalously high density of BOB is considered the most promising indicator for petroleum prospecting, as butane can only come from oil and gas reservoirs (Zhang et al., 2013).

Most of the previous studies have used culture-dependent methods to investigate the BOB in oil- and gas-contaminated environments (Zhang et al., 2013), which led to the isolation of Gram-positive CNMR (*Corynebacterium*, *Nocardia*, *Mycobacterium*, and *Rhodococcus*) strains and Gram-negative strains of *Thauera butanivorans* (McLee et al., 1972; Takahashi et al., 1980; Ashraf et al., 1994; Saunders et al., 1999; Cooley et al., 2009). These BOB can also utilize ethane and propane as carbon sources, but they cannot use methane (Zhang et al., 2013). As one of the greenhouse gas and a precursor to many atmospheric pollutants (such as alkyl nitrates and ozone) (Collins et al., 2002; Katzenstein et al., 2003), butane is emitted into the biosphere at a rate of 10 Tg per year via hydrocarbon seeps, mud volcanoes, and hydrothermal vents (Jaekel et al., 2014). Consequently, increasing concentrations of butane in the atmosphere is the cause of many environmental problems, such as global warming, ozone enrichment, and photochemical smog formation (Chan et al., 2006). Therefore, BOB are significant in both petroleum exploration and bioremediation. Nevertheless, the diversity and biogeography of BOB in oil and gas fields are still not sufficiently explored (Cappelletti et al., 2015).

Soluble di-iron monooxygenases (SDIMOs) are essential enzymes for diverse bacteria to initiate the oxidation of hydrocarbons (Coleman et al., 2006). Based on component arrangement, substrate specificity, and sequence similarity, SDIMOs are classified into aromatic/alkene monooxygenases (group 1), phenol monooxygenases (group 2), soluble methane monooxygenases (group 3), alkene monooxygenases (group 4), THF/propane monooxygenases (group 5), and an additional unclassified group (group 6) (Leahy et al., 2003; Coleman et al., 2006). The butane metabolism pathway in *Pseudomonas butanovora* ATCC43655 has been studied in detail (Arp, 1999). In this pathway, *n*-butane is first oxidized to butanol, then to butyraldehyde and finally to butyrate that can be directly assimilated into cell materials. This pathway is activated by butane monooxygenases (BMOs), which are non-heme iron monooxygenases (Sluis et al., 2002). The substrates for BMO usually range from C_2_ to C_9_ alkanes (Halsey et al., 2006; Doughty et al., 2008). The *bmoX* gene, encoding the alpha hydroxylase subunit of BMO (BmoX) classified into the group 3 SDIMOs (Coleman et al., 2006), is a conserved gene segment and its insertional mutation in a *P. butanovora* strain results in the loss of the ability to grow on butane (Sluis et al., 2002). Zhang Y. et al. (2016) reported that the expression level of *bmoX* genes can reflect the butane-oxidizing activity of BOB. Despite the importance of the *bmoX* gene in butane oxidation, its sequence diversity and distribution in BOB species have been rarely studied.

Isolation method is fundamental but most of the microorganisms in nature are uncultivable (Amann et al., 1995), therefore, this method underestimates the prokaryotic diversity and may ignore important microbes (Oren, 2004). Moreover, it is difficult to describe complex interactions within microbial communities in complex environments by means of isolation method (Jones et al., 2011). SIP, a culture-independent method, is a powerful tool for identifying active microorganisms that are able to metabolize specific substrates in complex systems (Radajewski et al., 2000). In this strategy, substrates are labeled with heavy isotopes, such as ^13^C and ^15^N, and used to feed microorganisms in environmental samples. The stable isotope is then assimilated into cellular components (such as DNA, RNA, and protein). Subsequently, the components labeled with heavy atoms are separated from the unlabeled biomass by density gradient centrifugation. Microbes participating in the metabolism of the labeled substrates can be identified and characterized by analyzing the labeled DNA (Gutierrez et al., 2013). In recent years, SIP has been widely used to identify bacteria that are capable of metabolizing alkane hydrocarbons and aromatic hydrocarbons (Song et al., 2015, 2016; Paul et al., 2017; Posman et al., 2017). However, the successful application of SIP to aerobic BOB has not been reported.

In the present study, to investigate the diversity of BOB in hydrocarbon-contaminated soils and the influence of butane on the soil microbial community, DNA-SIP was applied to Puguang gas field soils. In addition, the abundance of *bmoX* genes in butane-amended microcosms were determined by real-time quantitative PCR, and the diversity of *bmoX* genes were analyzed with clone libraries. The results provide more insight into BOB in oil and gas field environments and suggest the potential use of BOB in petroleum exploration and bioremediation.

## Materials and Methods

### Sample Collection

Soil samples were collected as mentioned previously (Deng et al., 2016). Briefly, gas field soils (G) with long-term microseepage were collected from a site adjacent to gas pumping wells at the Puguang gas field (31°31′48^′′^N, 107°46′12^′′^E) in Sichuan Province, China. Non-gas field soils (NG) served as background samples and were taken from sites (31°30′22^′′^N, 107°40′16^′′^E) that were approximately 10 km away from the gas field. Petroleum and gas reservoirs had never been discovered below the NG site. A geographical map indicating the sampling sites is provided in the Supplementary Figure S1. Considering the aerobic character of BOB and to avoid the effects of human cultivation, all the soils were taken from a depth of 30–50 cm in five replicates, then mixed thoroughly and transported to the laboratory in pre-sterilized plastic bags. The samples were stored at 4°C and –20°C for further analysis.

### Setup of Butane-Degrading Microcosms

Microcosms were constructed with 10 g of fresh soils in 50-ml pre-sterilized serum bottles. After sealing the bottles with butyl rubber stoppers and fixing the stoppers in place with aluminum crimp caps, 6 ml of air was taken out from the bottles with a gas-tight syringe (SGE, Australia), and then equal volume of unlabeled butane (99.99%, Beiwen Gas Manufacturer, Beijing, China) or ^13^C-butane (99%, CGN, Beijing, China) was injected into the bottles. Four treatments were prepared, including unlabeled butane-amended treatments (^12^C-BT), ^13^C-labeled butane-amended treatments (^13^C-BT), negative controls without butane addition (NB), and sterile controls with unlabeled butane addition (^12^C-ST). In addition, soils were autoclaved at 121°C for 30 min on three consecutive days to prepare sterile controls (Carter et al., 2007). Twelve serum bottles were prepared for each treatment and cultured in the dark at room temperature. Three of twelve were harvested at the desired time (6, 9, 12, and 14 days) and pooled, then they were stored at –20°C for DNA extraction. The residual butane in the microcosms was measured at 0, 6, 9, 12, and 14 days by gas chromatography–flame ionization detection as described in detail below.

### Butane Analysis

Previous studies have demonstrated that there are no significant differences in the removal rates of ^13^C-labeled and unlabeled substrates (Jiang et al., 2015; Song et al., 2015). Therefore, only the soil samples amended with unlabeled butane were selected to analyze the butane degradation in this study. To collect gas samples from microcosms, 10-ml penicillin bottles containing 2 ml of saturated sodium chloride solution were prepared. At 0, 6, 9, 12, and 14 days, 0.2 ml aliquots of gas (in triplicate) from each treatment were taken out from the top of the serum bottles and injected to the penicillin bottles using a gas-tight syringe. The penicillin bottles were inverted immediately to prevent the escape of butane and transported to the National Research Center for Geoanalysis at the Chinese Academy of Geological Sciences. The butane concentration in each penicillin bottle was determined by gas chromatography (Finnigan Trace GC Ultra; Thermo-Finnigan, Germany) equipped with a flame ionization detector and a 30-m length, 0.32-mm inner diameter column (Agilent HP-AL/S, Santa Clara, CA, United States) as described by Deng et al. (2016). Data were analyzed by unpaired two-tailed Student’s *t*-tests using the statistical software SPSS (SPSS, Inc., Chicago, IL, United States). The differences between treatments were considered significant at *p* < 0.05.

### DNA Extraction, Centrifugation, and Fractionation

Total genomic DNA was extracted from 0.5 g soil samples collected from ^12^C-BT, ^13^C-BT, and NB at each time point in duplicate using the E.Z.N.A. Soil DNA Kit (Omega, Norcross, GA, United States), according to the manufacturer’s protocol. The DNA concentration of each extract was determined using P300 Nanophotometer Spectrophotometers (IMPLEN, München, Germany). Then, DNA was stored at -20°C for further analysis.

Subsequently, the DNA of ^12^C-BT and ^13^C-BT were processed by density gradient centrifugation to separate the unlabeled and ^13^C-labeled DNA as previously described (Zheng et al., 2014). Briefly, approximately 2 μg DNA was mixed well with 4.9 ml of CsCl stock solution, and then gradient buffer (pH 8.0; 100 mM Tris–HCl; 100 mM KCl; and 1.0 mM EDTA) was added to a final volume of 5 ml. The average buoyant density (BD) of the mixture was determined with an AR200 digital hand-held refractometer (Reichert Inc., Buffalo, NY, United States), and adjusted to 1.725 g/ml using CsCl solution or gradient buffer, if necessary. The mixture was added to a 5.1 ml Quick-Seal polyallomer tubes and centrifuged at 190,000 *g* for 44 h at 20°C with a Vti 65.2 vertical rotor (Beckman Coulter, Inc., Palo Alto, CA, United States). After centrifugation, the DNA solution was fractionated into fifteen aliquots of 340 μl each using an NE-1000 single syringe pump (New Era Pump Systems, Inc., Farmingdale, NY, United States). The BD of each fraction was measured and the CsCl was removed by PEG precipitation (polyethylene glycol 6000). Finally, the DNA pellet was purified with 70% ethanol and dissolved in 30 μl sterile water.

### High-Throughput Sequencing and Data Analysis

The V3–V4 regions of bacterial 16S rRNA genes in unfractionated DNA of ^12^C-BT, ^13^C-BT, and NB, along with fractionated DNA from fractions 4 to 12 (the BD was between 1.712 and 1.737 g/ml) of each treatment, were amplified using the 340F/805R primer set. Sequencing of the amplicons was performed on the Illumina HiSeq platform by Beijing Microread Genetics Co., Ltd. (Beijing, China).

The acquired sequences were assigned to each sample with unique barcodes. Paired-end reads were merged using FLASH (Edgar, 2013), and filtered by Quantitative Insights Into Microbial Ecology (QIIME) to remove uncorrectable barcodes, primer mismatches, or ambiguous bases. Chimeric sequences were detected and removed as described previously (Edgar et al., 2011). Sequences were analyzed by the QIIME software package and the UPARSE pipeline, and assigned to operational taxonomic units (OTUs) based on 97% cutoff (Stackebrandt and Goebel, 1994; Edgar, 2013). Sequences with the highest abundance in each OTU were selected as representatives for the OTUs and annotated using the Greengenes database (vision 13.8). The relative abundance of each OTU was determined and the top 100 OTUs with high levels of relative abundance were selected for further analysis (Li et al., 2017). Butane degraders in samples were determined by OTUs enriched in the heavy fractions from ^13^C-BT compared with ^12^C-BT samples. In this study, three OTUs encoded as OTU 5, OTU 6, and OTU 49 were enriched in the butane-amended gas field samples, and these were aligned to *Giesbergeria* spp., *Pseudom*onas spp., and *Ramlibacter* spp., respectively.

Detrended correspondence analysis (DCA) of bacterial composition based on the Chi-square distance matrix was performed using the vegan package version 2.2-1 in the R computing environment. Because some DNA samples failed to be sequenced, only the samples with qualified sequences are presented in the Section “Results.”

### Real-Time Quantitative PCR of *bmoX* Genes and 16S rRNA Genes

Copy numbers of *bmoX* genes and 16S rRNA genes in unfractionated DNA of ^12^C-BT as well as fractionated DNA of ^13^C-BT and ^12^C-BT were determined by real-time quantitative PCR (qPCR). The primers for *bmoX* genes were bmoX-F: 5′-TGG TTC GAG CAC AAC TAY CCN GGN TGG-3′ and bmoX-R: 5′-TGC GGC TGC GCG ATC AGC GTY TTN CCR TC-3′ (Zhang et al., 2013). These primers were designed according to the conserved fragments among 2 *bmoX*, 6 *prmA*, 2 *prm1A*, and 5 *mmoX* sequences downloaded from the National Center for Biotechnology Institute (NCBI) database. They had been verified to amplify the *bmoX* genes from the standard butane-oxidizing bacterium *P. butanovora* ATCC43655 (Sluis et al., 2002). The primers for the 16S rRNA genes were 785F: 5′-GGA TTA GAT ACC CBR GTA GTC-3′ and 1061R: 5′-TCA CGR CAC GAG CTG ACG AC-3′ (Bogaert et al., 2011). The qPCR procedures for the *bmoX* genes and the 16S rRNA genes were the same except for the primers. Each PCR mixture contained 10 μl of SYBR *Premix Ex* Taq (TaKaRa Bio, Beijing, China), 0.2 μl of ROX Reference Dye II, 0.2 μl of each primer, 1 μl of DNA template, and 8.4 μl of deionized water in a final volume of 20 μl. The amplification reactions were performed in a 96-well plate on a QuantStudio 6 Flex System (Applied Biosystems) as follows: denaturation at 95°C for 30 s, followed by 40 cycles of 5 s at 95°C, 30 s at 60°C, and 30 s at 72°C, at which the SYBR green signal intensities were determined. After the PCR stage, melting curves were obtained by increasing the temperature from 60 to 95°C. To prepare the standard curve, *bmoX* genes amplified from the ^12^C-BT of gas field soils were ligated to the pGEM^®^-T vectors (Promega, Madison, WI, United States) and transformed into *E. coli* DH5α (Biomed, Beijing, China). A positive clone of *E. coli* containing *bmoX* genes was picked out, then the DNA of pGEM-T vectors was extracted from *E. coli* using the StarPrep Plasmid Miniprep Kit (GenStar, Beijing, China). The extract served as a standard DNA template. A decimal dilution series of the DNA template over five orders of magnitude was set up. The standard curve of 16S rRNA genes was made in a similar way to that of *bmoX* genes. All the DNA samples were run in triplicate and the average value was determined as the copy numbers for each sample. Data were analyzed by unpaired two-tailed Student’s *t*-tests using the statistical software SPSS (SPSS, Inc., Chicago, IL, United States). The differences between treatments were considered significant at *p* < 0.05.

### Cloning, Sequencing, and Phylogenetic Analysis of *bmoX* Genes

The *bmoX* genes in ^12^C-BT of gas field (G) samples harvested at each time point were amplified with the primers bmoX-F/bmoX-R described above. The 20-μl reaction mixture that included 10 μl of 2×Taq PCR StarMix (GenStar, Beijing, China), 0.2 μl of each primer, 2 μl of genomic DNA, and 7.6 μl of deionized water was run on a Veriti 96-well Thermal Cycler (Thermo Fisher Scientific, MA, United States) with the following conditions: initial denaturation at 95°C for 10 min, 35 cycles of repeated denaturation at 95°C for 30 s, annealing for 30 s (annealing temperature decreased by 0.5°C per cycle for 20 cycles, then held at 46°C for 15 cycles), and extension at 72°C for 1 min, followed by a final extension step at 72°C for 10 min. The PCR amplicons were gel purified using a Universal DNA Purification kit (TIANGEN Biotech, Beijing, China) and ligated into pGEM^®^-T vectors. The products were transformed into *E. coli* DH5α following the manufacturer’s guidelines. The transformed clones were spread on Luria-Bertani (LB) medium with ampicillin (50 μg/ml) for blue/white screening. The white strains were considered as positive clones and randomly picked to verify DNA insertion by PCR with M13F/M13R primers (Deng et al., 2017). Then, the positive clones with inserts were grown on LB plates for 12 h and sent to the Beijing Genomics Institute (Beijing, China) for sequencing. The sequences (vector sequences removed) were compared with the GenBank nucleotide database library via BLASTX. The deduced protein sequences obtained from the *bmoX* genes were grouped into operational protein families (OPFs) based on a cutoff value of 87% sequence identity. The α-diversity and coverage values were compared for all clone libraries at the cutoff levels of 83, 87, 90, and 95%, respectively. Finally, the value of 87% was selected. The representative sequences of all the OPFs that comprised not less than two sequences were aligned by ClustalW with their reference sequences for distinctive groups of SDIMOs, and a phylogenetic tree was reconstructed by MEGA 6.0 using a neighbor-joining method with 1000 bootstrap replications.

### Nucleotide Sequence Accession Number

The sequences obtained by Illumina HiSeq sequencing are available in the NCBI Sequence Read Archive (SRA) under the accession number SRP135746. The *bmoX* gene sequences used for tree construction were deposited in GenBank with accession numbers KY399888 to KY399891, KY399894, KY399898, KY399901, KY399902, KY399903, KY399907, KY399915, MG557980, MG557981, and MG557982.

## Results

### Butane Degradation in Microcosms

Detailed data on the butane biodegradation in the samples from the gas field (G) and non-gas field (NG) are shown in Supplementary Table S1. The residual butane was 86.0 and 75.4% in the sterile controls of the G and NG microcosms, respectively, after 14 days of incubation; these decreases in butane levels were regarded as being due to physical loss during the relatively long incubation time. Butane biodegradation was calculated from the difference between the total butane reduction and the physical loss in the microcosms. Significant butane biodegradation was observed in the unsterilized microcosms of the G samples, where 16.1, 19.9, and 20.7% butane were degraded after 9, 12, and 14 days of incubation, respectively. In contrast, no significant butane biodegradation was found in the unsterilized NG microcosms.

### Microbial Community Structure Analysis After Butane Incubation

**FIGURE 1:**
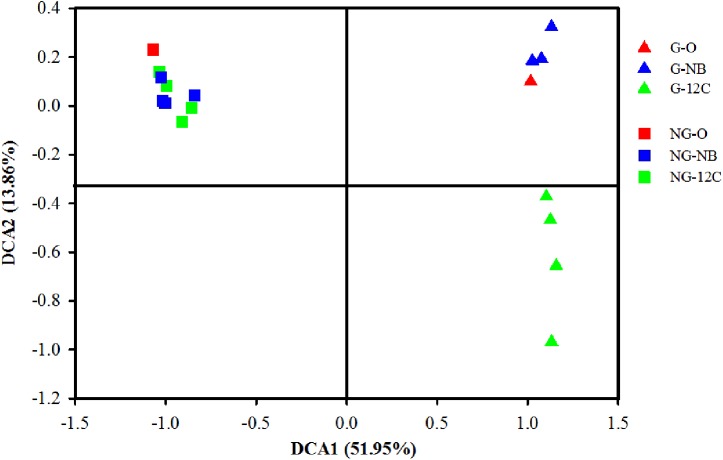
Detrended correspondence analysis (DCA) of bacterial community structures of ^12^C-BT and NB samples. G, samples from gas field; NG, samples from non-gas field; O, original samples without incubation; NB, incubated without butane; 12C, incubated with ^12^C-butane.

To study the effects of butane on the bacterial community structures in the soil samples, DNA extracted from the samples amended with unlabeled butane (^12^C-BT) and negative controls incubated without butane (NB) were subjected to sequencing before centrifugation and fractionation, and then, DCA was conducted. DCA axis 1 explains 51.95% of the variability, and DCA axis 2 explains 13.86%, with a cumulative percentage of 65.81% (**Figure 1**). The DCA plot shows a clear separation between the G samples and NG samples, indicating that their bacterial community structures are different from each other. The G samples incubated with ^12^C-butane rapidly deviate from the original sample and cluster in another location of the DCA plot, which suggests an early alteration in the microbial community. There was a slight change of the microbial community of the NG samples after incubation with butane, which is reflected by their grouping around the original NG sample. These results demonstrate that butane can rapidly shape the microbial community of gas field soils that undergo long-term microseepage, but butane has limited effects on non-gas field soils within a short period of time.

**FIGURE 2:**
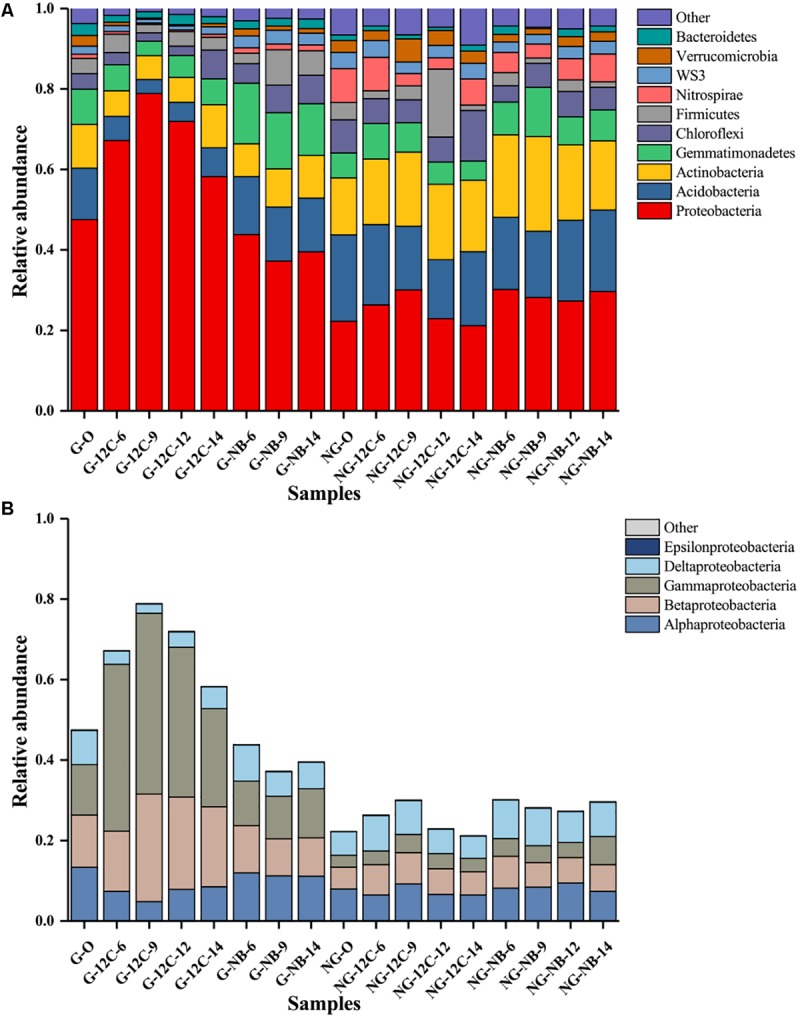
Relative abundance of the top 10 phyla **(A)** and proteobacterial classes **(B)** in samples based on 16S rRNA genes sequences. G, samples from gas field; NG, samples from non-gas field; O, original samples without incubation; NB, incubated without butane; 12C, incubated with ^12^C-butane.

The relative abundance levels of the top 10 phyla in all of the samples are shown in **Figure 2A**. Proteobacteria was the most predominant phylum in all samples, and its relative abundance in the original G sample was 47.5%, twice as high as that in the original NG sample (22.3%). Notably, the relative abundance of Proteobacteria in the G samples sharply increased in the early days and reached the highest value at day 9 (78.9%), then it showed a slight decrease at day 12 and 14. As to the members of the Proteobacteria, only Betaproteobacteria and Gammaproteobacteria were enriched in the G samples after incubated with butane (**Figure 2B**). The relative abundance of Betaproteobacteria in the G samples increased by 1.9, 13.8, 10.0, and 6.9% after incubated with butane for 6, 9, 12, and 14 days, respectively. The relative abundance of Gammaproteobacteria in the G samples had a greater change, increasing by 29.0, 32.4, 24.8, and 11.9% after incubated with butane for 6, 9, 12, and 14 days, respectively. These results suggest that Betaproteobacteria and Gammaproteobacteria play important roles in the early stage of butane degradation. However, only slight modifications in the relative abundances of all phyla were observed in the NG samples and negative controls, which was consistent with the results of the DCA (**Figure 1**).

### BOB in Soil Samples

The 16S rRNA genes in each fraction of the ^12^C-BT and ^13^C-BT samples were quantified, and the results are shown in Supplementary Figure S2. An apparent shift to higher BD was observed in the ^13^C-BT compared to ^12^C-BT of the G samples after incubation for 12 days, and the increase of ^13^C-labeled 16S rRNA genes (in the heavy fractions) at day 12 and 14 in G was significant (*p* < 0.05) in comparison with those at day 6 and 9 in the same samples and those in the NG samples, indicating that ^13^C-butane was assimilated by BOB in the G samples.

**FIGURE 3:**
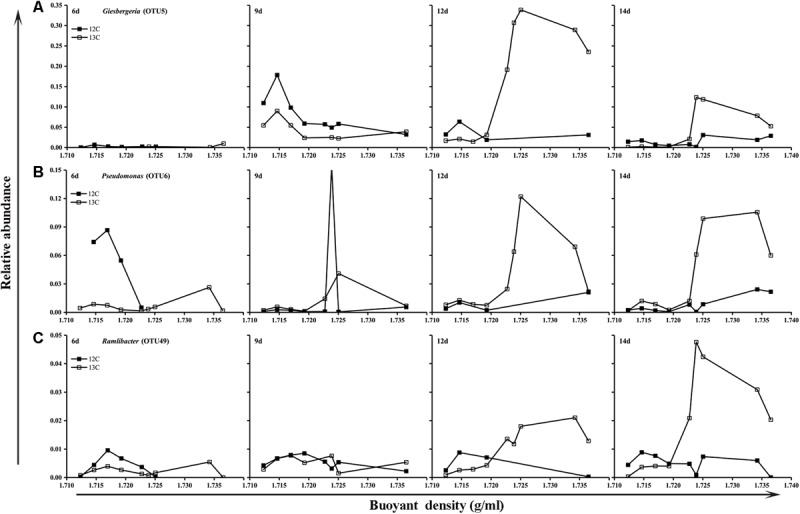
Relative abundance of *Giesbergeria* **(A)**, *Pseudomonas* **(B)**, and *Ramlibacter* **(C)** against the buoyant density gradients in gas field samples (G).

The bacteria responsible for butane assimilation were determined by comparing the relative abundances of specific microorganisms represented by OTUs in the fractions of the ^13^C-BT and ^12^C-BT samples. The results showed that OTU 5 was enriched at 12 and 14 days in the G microcosms supplied with ^13^C-butane, and its relative abundance at heavy buoyant densities (>1.723 g/ml) in the ^13^C-BT samples greatly exceeded that in the ^12^C-BT samples at day 12 and 14 (**Figure 3**). Similarly, the relative abundance levels of OTU 6 and OTU 49 at the heavy BDs were also higher in the ^13^C-BT samples than in the ^12^C-BT samples at 12 and 14 days. Compared to the ^12^C-BT samples, the enrichment of OTU 5, OTU 6, and OTU 49 in the heavy fractions of the ^13^C-BT samples indicated that bacteria represented by these OTUs played a key role in butane oxidation. However, these OTUs were not enriched at heavy BDs in either the ^13^C-BT or ^12^C-BT of the NG samples (Supplementary Figure S3). Using the Greengenes database, OTU 5, OTU 6, and OTU 49 were matched to *Giesbergeria* spp., *Pseudom*onas spp., and *Ramlibacter* spp., respectively.

### Occurrence of *bmoX* Genes

**FIGURE 4:**
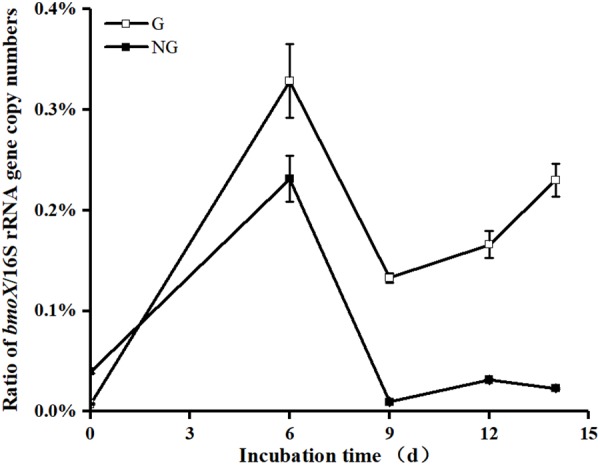
Comparison showing the significantly greater (*p* < 0.05) ratio of gene copy numbers of *bmoX/*16S rRNA gene in unfractionated DNA from gas field soil samples (G) than that from non-gas field soil samples (NG) after incubated with butane for 9, 12, and 14 days. Data are averages of three replicates. Error bars represent SD, some error bars are smaller than the symbols.

**FIGURE 5:**
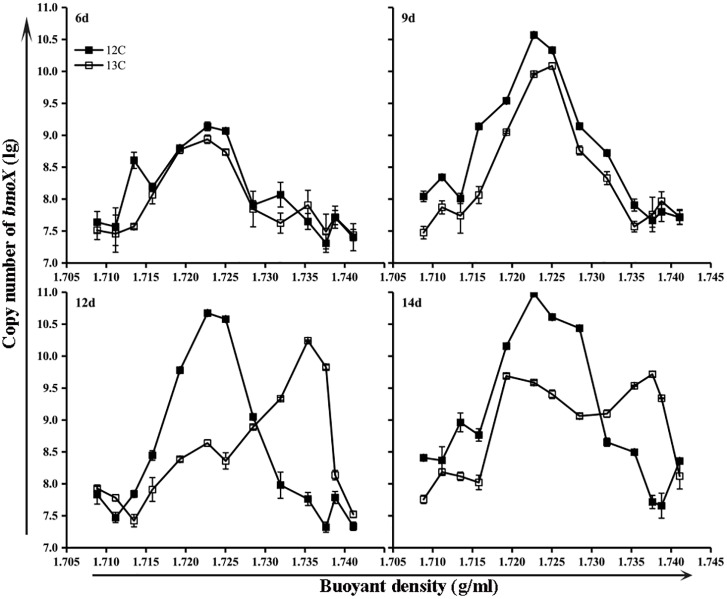
Gene copies of *bmoX* genes (lg) in fractions of gas field soil samples (G) incubated with ^12^C-butane and ^13^C-butane showing that the *bmoX* genes were labeled after 12 days of incubation, since the *bmoX* gene numbers of the ^13^C-BT samples were significantly increased (*p* < 0.05) in the heavy fractions (1.7354 and 1.7377 g/ml) at day 12 and 14 in comparison with those at day 6 and 9. Data are averages of three replicates. Error bars represent SD, some error bars are smaller than the symbols.

The *bmoX* genes in the unfractionated G and NG samples were quantified by real-time quantitative PCR. To minimize the effects of environmental disturbance in soil samples, we normalized the abundance of the *bmoX* genes to the total abundance of the 16S rRNA genes, and the absolute copy numbers of the *bmoX* genes and 16S rRNA genes are shown in Supplementary Figures S4, S5, respectively. The results (**Figure 4**) show that the abundance of the *bmoX* genes increased in the gas field soils after incubation with butane, as the ratio increased by 43.14-, 17.39-, 21.74-, and 30.14-fold at days 6, 9, 12, and 14, respectively. In contrast, the *bmoX* genes in the non-gas fields samples showed a slight increase at day 6 (by 5.80-fold), followed by decreases at day 9 (by 0.76-fold), day 12 (by 0.21-fold), and day 14 (by 0.42-fold). Thereafter, the *bmoX* genes in each fraction were also quantitatively analyzed. For the G samples (**Figure 5**), the majority of *bmoX* genes in the ^12^C-BT samples were found in the light fractions (BD < 1.7285 g/ml) at 6, 9, 12, and 14 days, and the highest point (BD ≈ 1.7227 g/ml) remained the same during the incubation process. In the ^13^C-BT samples, the *bmoX* genes were also concentrated in the light fractions at day 6 and 9, and these results were identical to those of the ^12^C-BT samples. However, after being incubated for 12 and 14 days, the *bmoX* genes were significantly transferred to the heavy fractions (BD > 1.7319 g/ml), and the copy numbers of the *bmoX* genes reached the highest point at the BD values of 1.7354 and 1.7377 g/ml, suggesting that the *bmoX* genes were labeled and spundown during the isopycnic ultracentrifugation of the total DNA. For the NG samples, there were no significant differences between the ^13^C-BT and ^12^C-BT in the whole incubation process (Supplementary Figure S6).

### Phylogenetic Characterization of *bmoX* Genes

**FIGURE 6:**
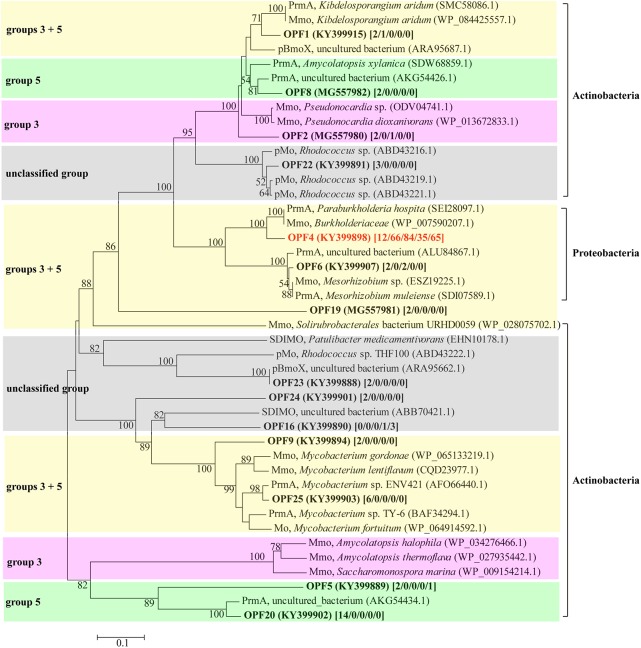
Phylogenetic tree of representative deduced *bmoX* sequences generated from different clone libraries of gas field soil samples (G) and reference sequences from GenBank. The tree was created using the neighbor-joining method with 1000 bootstrap replications; bootstrap values over 50% are shown at the branch nodes; the scale bars represents 10% sequence divergence. The GenBank accession number of all sequences used to build the tree is given in parentheses. The number of clones for each OPF is given in brackets [G-0/G-6/G-9/G-12/G-14]. Abbreviations for SDIMOs were as follows: Mmo, methane monooxygenase; PrmA, propane monooxygenase hydroxylase large subunit; pBmoX, putative butane monooxygenase alpha subunit; Mo, monooxygenase; pMo, putative monooxygenase. Sequences in clusters with pink background belong to group 3 SDIMOs; sequences in clusters with green background belong to group 5 SDIMOs; clusters with yellow background include both sequences belonging to group 3 and group 5 SDIMOs; clusters with gray background are unclassified SDIMOs and monooxygenases.

To study the diversity of the *bmoX* genes in the G samples at the phylogenetic level, five clone libraries (G-0, G-6, G-9, G-12, and G-14) were constructed to represent samples from each time point and 330 sequences were obtained. Both the asymptotic behavior of the rarefaction curves (Supplementary Figure S7) and the coverage values (**Table 1**) of all libraries indicated a satisfactory sampling effort. The number of OPFs (87% sequence similarity cutoff), the α-diversity, and OPF richness (Chao 1) were calculated for each sample (**Table 1**). The highest and lowest numbers of observed OPFs were 22 in sample G-0 and 2 in G-12, respectively. Interestingly, the α-diversity and richness decreased sharply at day 6, and then, both rose again at day 14. The deduced protein sequences of all of the OPFs represented by two or more sequences were selected to construct the phylogenetic tree (**Figure 6**). The deduced protein sequences of the *bmoX* genes in this study had 60–98% similarities with the sequences within group 3 SDIMOs, group 5 SDIMOs, and unclassified monooxygenases from Alphaproteobacteria, Betaproteobacteria, and Actinobacteria. Most of the clones (79.4%) were defined as OPF4 that showed high affinity to methane monooxygenase (Mmo-like) from *Burkholderiaceae* (WP_007590207, 90%) and propane monooxygenase hydroxylase large subunit (PrmA-like) from *Paraburkholderia hospital* (SEI28097.1, 90%). The G-0 sequences were distributed over 13 OPFs, such as OPF4, OPF25 with 98% identity to PrmA-like sequences from *Mycobacterium* sp. ENV421 (AFO66440), and OPF20 matched to PrmA-like sequences from uncultured bacteria (AKG54434, 94%). Almost all clones of G-6, G-9, G-12, and G-14 were clustered into the OPF4, indicating that incubation with butane reduced the diversity of *bmoX* genes in the samples.

**Table 1:** The diversity indexes and coverage of *bmoX* gene clone libraries of G samples incubated with ^12^C-butane for 0, 6, 9, 12, and 14 days (abbreviated as G-0, G-6, G-9, G-12, and G-14, respectively).

Sample	*N*	OPF numbers	Chao1	H’	C
G-0	66	21	26	3.80	0.86
G-6	68	3	4	0.22	0.97
G-9	87	3	3	0.24	0.99
G-12	36	2	2	0.18	0.97
G-14	73	7	17	0.76	0.93


*N, number of clones; OPF numbers, observed OPF numbers (87% sequence similarity cutoff); Chao1, Chao1 richness estimate; H’, Shannon index of diversity; C, coverage.*

## Discussion

Revealing the bacteria related to butane metabolism and their responses to light hydrocarbon microseepage are important for the environment and for microbial ecology. However, the characterization of BOB and the quantification of their functional genes in oil and gas fields *in situ* are still challenging. In the present study, we analyzed BOB in soils from Puguang gas field by DNA-SIP; additionally, the expression and diversity of *bmoX* genes were investigated.

Long-term microseepage of gaseous hydrocarbons may lead to anomalous enrichment of alkane-oxidizing bacteria, which causes the microbial community structure in the surface soil of oil and gas reservoirs to be different from those in non-oil and gas soils (Wu et al., 2014; Deng et al., 2016). In this study, the clear separation of the samples from the gas field and the non-gas field in the DCA plot indicates the great disparity of their microbial community structures (**Figure 1**), which is similar to the results of previous studies (Xu et al., 2013a,b). The rapid change in the microbial community in the butane-incubated G samples, but not the NG samples (**Figure 1**), demonstrates that BOB already exist in the G samples as microorganisms adapted well to a long-term and continuous supply of light hydrocarbons. Thus, they can rapidly respond to butane input. An anomalously high abundance of hydrocarbon-oxidizing bacteria (HOB) in the surface soil is usually used as an indicator for the MPOG (Rasheed et al., 2012; Veena Prasanna et al., 2013), and a good connection between the HOB abundance and the existence of oil and gas reservoirs has been observed (Rasheed et al., 2013). Additionally, the abundance levels of n-alkane-degrading bacteria in oil and gas soil samples are significantly higher than those in background soils (Xu et al., 2013a). Although our results showed an obvious microbial dissimilarity between gas field and non-gas field soils, whether MPOG can be applied in the Puguang gas field still requires further evidence from more samples.

The predominance and dramatic increase in the relative abundances of Proteobacteria, especially Betaproteobacteria and Gammaproteobacteria, in the G samples at day 6 and 9 (**Figures 2A,B**) suggests that Betaproteobacteria and Gammaproteobacteria might be mainly responsible for degradation in the early stage of butane degradation. The slight decrease in their relative abundances in the G samples at day 12 and 14 might be due to the gradual occupation of the niche by other slow-response butane degraders or other bacteria coexisting with BOB in the consortium. Some bacteria from Betaproteobacteria and Gammaproteobacteria are capable of degrading short-chain hydrocarbons (Redmond et al., 2010), as well as other alkanes (Wallisch et al., 2014) and arenes (Deng et al., 2017), and they have also been detected in other microseepage ecosystems, such as the sedimentary basin in Brazil (Miqueletto et al., 2011). The identification of three OTUs belonging to the genera *Pseudomonas*, *Giesbergeria*, and *Ramlibacter* by the coupling of DNA-SIP and high-throughput sequencing (**Figure 3**) further proved the important roles of Betaproteobacteria and Gammaproteobacteria in butane biodegradation.

Gammaproteobacteria species from the genus *Pseudomonas* is widespread in the natural environment and is able to metabolize a wide range of organic compounds (Salgado-Brito et al., 2007; Dong et al., 2015; Baruah et al., 2016; Hemidouche et al., 2016), including light hydrocarbons (Zhang Y. et al., 2016). For this reason, *Pseudomonas* was entrusted with the task of biodegrading hydrocarbon pollutants in contaminated sites, as well as serving as a biological indicator of oil and gas reservoirs. Deng et al. (2016) proposed *Pseudomonas* as a universal biomarker for subterranean oil deposits because its relative abundance remarkably increased after being incubated with butane. Our results provide firm evidence for the degradation of butane by *Pseudomonas*-related bacteria in the soil of the Puguang gas field using DNA-SIP.

The identification of *Giesbergeria* and *Ramlibacter* as BOB by DNA-SIP in this study is a novel finding. The genus *Giesbergeria* was first defined by Grabovich et al. (2006), and only five species have been reported to belong to this genus, with the species *Giesbergeria voronezhensis* isolated from an active sludge^1^. Genus *Ramlibacter* was first described by Heulin et al. (2003), and four species in this genus have been reported^2^. Its species *Ramlibacter tataouinensis* was made famous for its morphological transition capacity (Gommeaux et al., 2005). Both *Giesbergeria* and *Ramlibacter* belong to Burkholderiales, an order in Betaproteobacteria with many members that are able to degrade hydrocarbons. Abbasian et al. (2016) reported that Bacillales, Flavobacteriales, Pseudomonadales, and Burkholderiales are enriched in crude oil-contaminated soil. Species within Burkholderiales and Sphingobacteriales are the pivotal benzo[*a*]pyrene degraders in Mt. Maoer soils (Song et al., 2015). Clone libraries of the *bmoX* gene in this study also indicate that some bacteria of the family *Burkholderiaceae* (order Burkholderiales) might be responsible for butane degradation (**Figure 6**). However, previous studies have not reported that *Giesbergeria* and *Ramlibacter* directly participate in butane oxidation because they have not been associated with butane metabolism previously. Our results prove for the first time that some representatives within these two genera are primarily responsible for butane oxidation in light hydrocarbon microseepage soil. The data presented in **Figure 6** revealed the diversity of *bomX* genes amplified in this study in both the SDIMO groups and the phylogenetic lineages, and demonstrated the inconsistence among the groups of SDIMOs and their sequence phylogeny, because the enzymes in the same group of SDIMOs, like those in groups 3 and 5, were divided into different phylogenetic lineages. The two unclassified lineages might imply the existence of novel SDIMOs and supported the suggestion of group 6 for SDIMOs by Coleman et al. (2006) since a group 6 SDIMO (ABB70421.1) was included in one of the unclassified lineage.

The *bmoX* genes are extremely important in butane oxidation (Sluis et al., 2002). We also found that *bmoX* genes were associated with butane degradation in G samples, as the increased abundance of *bmoX* genes in the G samples (**Figure 4**) was positively correlated with the significant butane degradation taking place in the G microcosms (Supplementary Table S1). Zhang Y. et al. (2016) observed a similar phenomenon, i.e., *bmoX* genes were upregulated as the butane degradation rate increased. High concentrations of oil might be toxic to microbes (Samanta et al., 2002), and therefore, many studies have reported that oil contamination decreases the diversity of microorganisms and the relevant functional genes in a variety of environments (Liang et al., 2011; Bell et al., 2013; Yang et al., 2014). In this study, we also found that the diversity of *bmoX* genes decreased after the microcosms were incubated with butane (**Table 1**). In contrast, higher bacterial diversity was found by Deng et al. (2016) in next-to-well samples polluted by trace petroleum hydrocarbons, compared to the background area. This difference indicates that the effects of pollutants on bacterial communities might vary depending on the hydrocarbon concentrations or soil features.

Previous studies have suggested that light hydrocarbon-oxidizing-genes, such as *pmoA* and *prmA*, could also be utilized as biological indicators for MPOG (Zhang C.Y. et al., 2016). With our results, BOB were enriched by adding butane in the G samples, but not in the NG samples (**Figure 3** and Supplementary Figure S3), while *bomX* richness was increased in both soil samples by adding butane (**Figure 4** and Supplementary Figure S4). These data might indicate that some *bomX*-harboring bacteria exist in NG soil, which could be enriched by adding butane. However, they are not BOB since no significant butane removal was detected in the corresponding sample. Therefore, *bomX* or its homologs may be also included in other metabolic pathways, other than butane oxidization. Before these issues are clarified in further studies, it is premature to use *bmoX* as indicator gene for oil and gas exploration. The first step in butane removal is the oxidization of butane into butanol by monooxygenases, therefore, the accumulation of butanol inhibited butane degradation. Moreover, the degradation ability of BOB is related to their tolerance to butanol (Zhang Y. et al., 2016). The decrease of *bomX* abundance in NG sample after 6 days of incubation (Supplementary Figure S4) might be explained by the possibility that butane stimulated the growth of *bomX*-harboring bacteria in NG sample, which oxidize the butane into butanol. However, these bacteria may have relatively low butane-degrading ability and low tolerance to butanol. Therefore, the accumulation of butanol inhibited their further increase. To confirm this estimation, further study on detection of butanol in the microcosm is needed. In addition, the persistence of high abundance of *bomX* in G samples is consistent with the enrichment of BOB and confirmed their participation in butane degradation.

An unexpected result in this study was the different microbial characterization results that were obtained from 16S rRNA genes analysis compared with *bmoX* genes analysis. Studies on BOB and *bmoX* genes have been relatively rare compared to other light HOB reported. Previously amplified *bmoX* genes have been detected in *P. butanovora* that is able to grow with butane (Sluis et al., 2002) and in a butane-oxidizing bacterium *Arthrobacter* sp. PG-3-2 isolated from the Puguang gas field (Zhang et al., 2013). Since there are only few *bmoX* gene sequences in the NCBI database^3^ (Sluis et al., 2002; Brzostowicz et al., 2005), we were unable to assign exact species identities to the obtained *bmoX* gene sequences. Moreover, the *bmoX* gene primers used in this study were designed with reference to the homologous SDIMOs-encoding genes of *bmoX* genes (Zhang et al., 2013), therefore some sequences generated by the clone libraries in this study might not be the genes for butane degradation. Horizontal gene transfer, which allows bacteria acquire exogenous genes from foreign species, is necessary for bacteria to survive in and adapt to harsh environmental conditions (Lawrence, 1997; Molbak et al., 2003). It has been pointed that horizontal gene transfer plays a vital role in the bioremediation of soils contaminated with petroleum hydrocarbons (Shahi et al., 2017). Li et al. (2017) also found that the phenanthrene degraders revealed by the 16S rRNA genes and the polycyclic aromatic hydrocarbons-ring hydroxylating dioxygenase (PAH-RHD) genes were different, and they speculated that horizontal gene transfer and hybridization might have occurred. Considering the high sequence similarity and homology between the *bmoX* genes and other SDIMOs-encoding (especially to methane monooxygenase and propane monooxygenase) genes, they may be recombined and shared extensively among different genera via horizontal gene transfer.

## Conclusion

This is the first study to utilize DNA-SIP for detecting the bacteria responsible for butane oxidation in gas field soils. Proteobacteria species from the classes Betaproteobacteria and Gammaproteobacteria were primarily responsible for butane oxidation in the Puguang gas field samples. *Pseudomonas*, *Giesbergeria*, and *Ramlibacter* were further proven to be the main BOB in gas field soils; the butane-degrading abilities of *Giesbergeria* and *Ramlibacter* are a novel observation. In addition, the diversity of *bmoX* genes in the G samples reduced after incubated with butane. These results expand our knowledge on BOB in light hydrocarbon-contaminated environments and provide valuable guidance for petroleum exploration and bioremediation.

## Author Contributions

YD, CD, and HY conceived and designed the experiments. YD performed all the experiments and analysis and wrote the paper. JY and BL participated in the soil samples collection. EW revised the manuscript. HY supervised the overall work, discussed the results, and revised the manuscript. All authors read and approved the final version of the manuscript.

## Conflict of Interest Statement

The authors declare that the research was conducted in the absence of any commercial or financial relationships that could be construed as a potential conflict of interest.

## Footnotes

1 http://www.bacterio.net/giesbergeria.html

2 http://www.bacterio.net/ramlibacter.html

3 https://www.ncbi.nlm.nih.gov/nuccore/?term=bmox

## ABBREVIATIONS

BOB: butane-oxidizing bacteria
DNA-SIP: DNA-based stable isotope probing
MPOG: microbial prospecting of oil and gas
SDIMO: soluble di-iron monooxygenase
