# Supplementary material for: Novel Butane-Oxidizing Bacteria and Diversity of bmoX Genes in Puguang Gas Field
Source: Front Microbiol. 2018 Jul 17;9:1576. doi: 10.3389/fmicb.2018.01576 (PMC6056644; doi:10.3389/fmicb.2018.01576)
Supplement: Supplementary file 1 [file Table_1.docx]

Supplementary Material

Novel Butane-Oxidizing Bacteria and Diversity of *bmoX* Gene in Puguang Gas Field

Yue Deng^1^, Chunping Deng^1^, Jinshui Yang^1^, Baozhen Li^1^, Entao Wang^2^, Hongli Yuan^1*^

*** Correspondence:** Hongli Yuan: [hlyuan@cau.edu.cn](mailto:hlyuan@cau.edu.cn)

# Supplementary Tables

Table S1 Butane remaining in microcosm over time ^a^

| Time (d) | G (ppm) | | | | NG (ppm) | | | | |
| --- | --- | --- | --- | --- | --- | --- | --- | --- | --- |
|  | ^12^C-ST | R (%) | ^12^C-BT | R (%) |  | ^12^C-ST | R (%) | ^12^C-BT | R (%) |
| 0 | 1866 ± 182 | 100 | 1887 ± 30 | 100 |  | 1897 ± 141 | 100 | 1944 ± 237 | 100 |
| 6 | 1734 ± 106 | 92.9 | 1690 ± 11 | 89.6 |  | 1642 ± 102 | 86.5 | 1756 ± 77 | 90.3 |
| 9 | 1765 ± 114 | 94.6 | 1483 ± 3 | 78.6^*^ |  | 1629 ± 30 | 85.9 | 1643 ± 50 | 84.5 |
| 12 | 1715 ± 137 | 91.9 | 1361 ± 23 | 72.1^**^ |  | 1620 ± 101 | 85.4 | 1557 ± 74 | 80.1 |
| 14 | 1605 ± 132 | 86.0 | 1230 ± 123 | 65.2^*^ |  | 1431 ± 85 | 75.4 | 1490 ± 118 | 76.6 |

^a^ G, soil samples from gas field; NG, soil samples from non-gas field; ^12^C-ST, the abiotic sterile treatment; ^12^C-BT, the treatment with ^12^C-butane as the sole carbon source. ^*^: Significantly lower than the control. Significance: ^*^, *p* < 0.05; ^**^, *p* < 0.001.

# Supplementary Figures


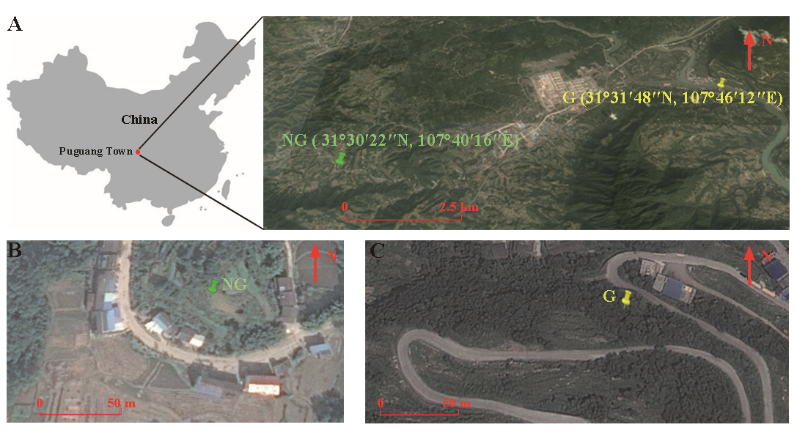


**Figure S1** Geographical map of sampling sites. G, the site for gas field samples; NG, the site for non-gas field samples. A, relative positions of the G and NG sites. B, magnification of NG site. C, magnification of G site.


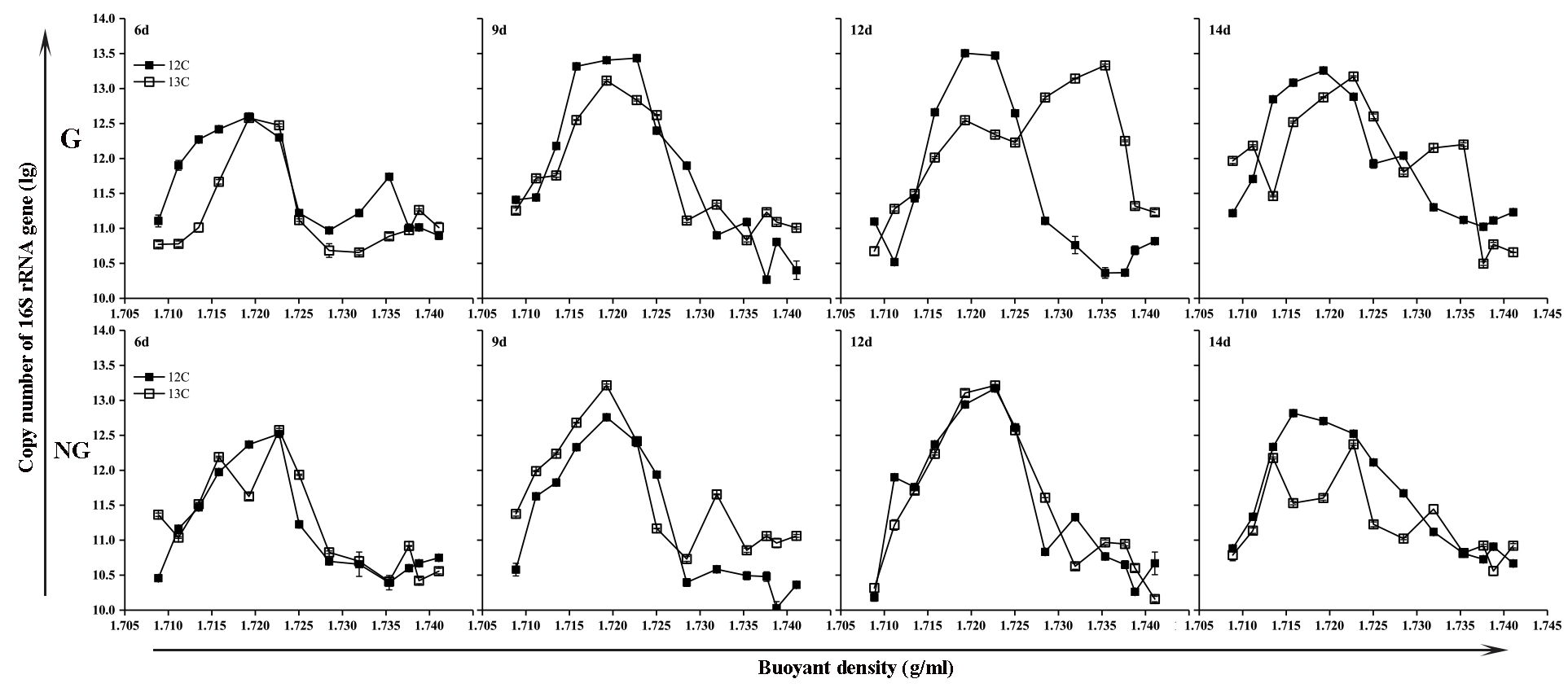


**Figure S2** Gene copies of 16S rRNA genes (lg) in fractions of soil samples incubated with ^12^C-butane and ^13^C-butane. G, samples from gas field; NG, samples from non-gas field. Data are the average of three replicates. Error bars represent standard deviation; some error bars are smaller than the symbols. The increase of ^13^C-labeled 16S rRNA genes (in the heavy fractions) at day 12 and 14 in G was significant (*p* < 0.05) in comparison with those at day 6 and 9 in the same sample and those in the NG sample.


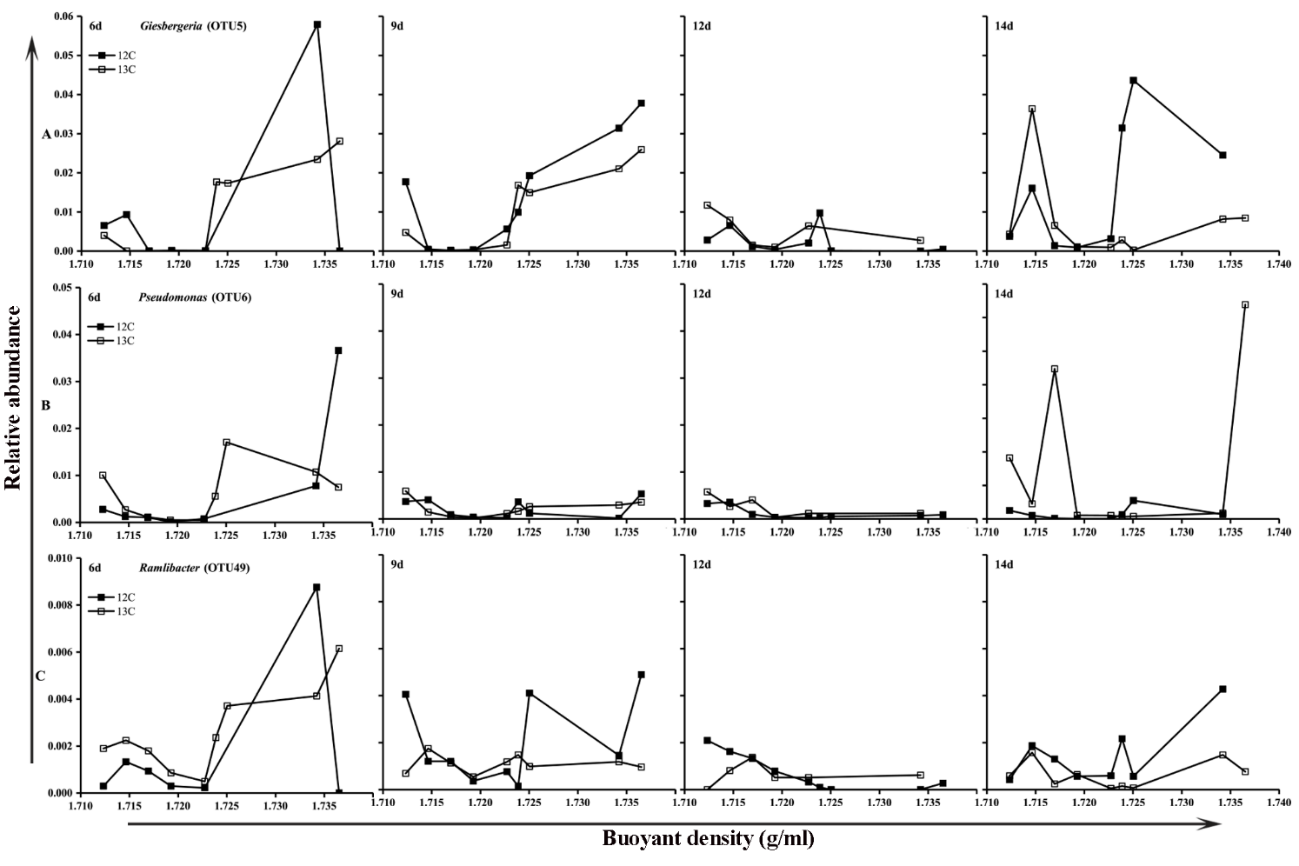


**Figure S3** Relative abundance of *Giesbergeria* (A), *Pseudomonas* (B) and *Ramlibacter* (C) against the buoyant density gradients in non-gas field (NG) samples.





**Figure S4** Gene copies of *bmoX* genes (lg) in un-fractionated DNA of gas field soil samples (G) and non-gas field soil samples (NG) incubated with ^12^C-butane. Data are averages of three technical replicates. Error bars represent standard deviation; some error bars are smaller than the symbols. The copy numbers of *bmoX* were significantly greater in NG sample than that in G sample at beginning and at day 6 and they were reverse for longer incubation time (9 to 14 days) (*p* < 0.05).





**Figure S5** Gene copies of 16S rRNA genes (lg) in un-fractionated DNA of gas field soil samples (G) and non-gas field soil samples (NG) incubated with ^12^C-butane. Data are averages of three technical replicates. Error bars represent standard deviation; some error bars are smaller than the symbols. Significantly greater copy numbers of 16S rRNA genes (*p* < 0.05) were observed in G after 9 days of incubation in comparison with the initial and 6 days of incubation with butane. While this change in NG was not significant (*p* > 0.05).


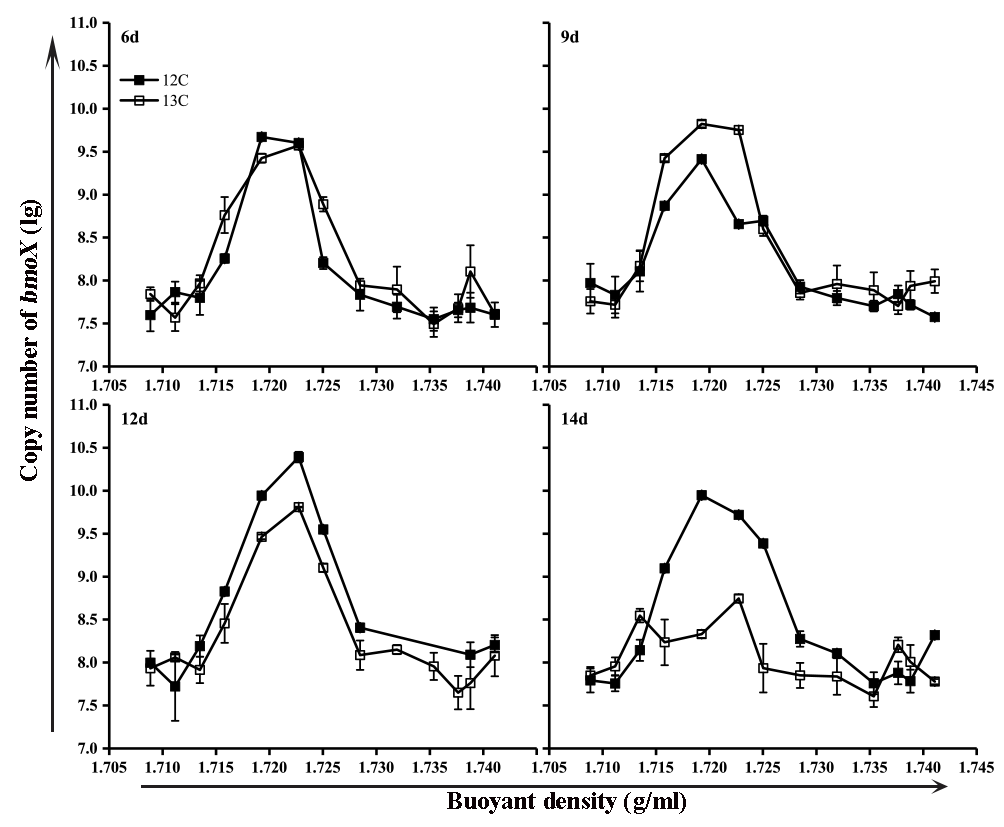


**Figure S6** Copy numbers of *bmoX* genes (lg) in fractions of non-gas field soil samples (NG) incubated with ^12^C-butane and ^13^C-butane. Data are the average of three replicates. Error bars represent standard deviation; some error bars are smaller than the symbols.





**Figure S7** Rarefraction curves of the deduced amino acid sequences of the *bmoX* genes retrieved from clone libraries of G samples incubated with ^12^C-butane for 0, 6, 9, 12 and 14 days (abbreviated as G-0, G-6, G-9, G-12 and G-14, respectively). The OPFs were defined using an 87% sequence identity cutoff.
